# Prevalence and factors associated with intention to use e-cigarettes among lower secondary school students in Bangkok: A cross-sectional study

**DOI:** 10.18332/tid/218847

**Published:** 2026-05-06

**Authors:** Saroj Nakju, Sunaree Tanapek, Supattra Assawamaitree, Kewarin Nitikorn

**Affiliations:** 1 Faculty of Public Health, Ramkhamhaeng University, Bangkok, Thailand

**Keywords:** intention to use e-cigarettes, e-cigarette smoking, lower secondary school students, theory of planned behavior

## Abstract

**INTRODUCTION:**

E- cigarettes among youth represent a global public health challenge. The rising prevalence of e-cigarette use among students is a significant public health issue in Thailand. Therefore, this study aimed to identify the factors associated with the intention to smoke e-cigarettes among lower secondary school students in the Bangkok metropolitan area, using the Theory of Planned Behavior (TPB).

**METHODS:**

A cross-sectional study was conducted among 300 students selected via two-stage cluster random sampling. Data were collected via a questionnaire between May and July 2024. Data analysis included descriptive statistics, chi-squared tests, and independent t-tests for bivariate analysis. Multivariable factors associated with intention were identified using binary logistic regression.

**RESULTS:**

In all, 90.7% of students had a low level of intention to use e-cigarettes. In the bivariate analysis, gender was significantly associated with the intention to use e-cigarettes (p=0.026). The binary logistic regression model, which adjusted for gender and all three TPB constructs, revealed that attitude towards e-cigarette prevention was the only significant factor associated with the intention to use e-cigarettes. Students with a positive attitude toward prevention were 3.82 times more likely to report a low intention to use e-cigarettes (AOR=3.82; 95% CI: 1.40–10.39).

**CONCLUSIONS:**

Attitude toward e-cigarette prevention is the primary factor associated with the intention to use e-cigarettes among lower secondary students. These findings suggest that focusing on a positive attitude and refusal self-efficacy, through school and community collaboration, may effectively support prevention efforts.

## INTRODUCTION

Tobacco use is a major global risk factor and a leading cause of chronic disease morbidity and mortality. The World Health Organization (WHO) estimates that tobacco-related diseases will account for 8 million deaths worldwide annually by 2030, with traditional cigarette smoking directly causing cardiovascular diseases, such as coronary artery narrowing and myocardial infarction^1^. In Thailand, while national forecasts suggested a slight decrease in the smoking rate among individuals aged ≥15 years (from 20.3% in 2014 to 17.5% by 2025), this reduction falls short of the United Nations’ global target of a 30% reduction by 2025. Furthermore, the average age of first tobacco use remains critically low, often occurring before the age of 10 years^2^.

Despite the overall decline in traditional smoking rates, the emergence of e-cigarettes poses a new and significant public health challenge, particularly among youth worldwide^3^. In Thailand, data from the National Statistical Office^2^ also indicated that 0.14% of the Thai population (78742 individuals) used e-cigarettes, with over half of them concentrated in the youth aged 15–24 years demographic in the Bangkok and central regions. This trend is alarming, as studies reveal that e-cigarette use among Thai youth has increased by 18%^4^. This increase is fueled by aggressive marketing that often promotes misinformation, claiming e-cigarettes are less harmful. However, compelling scientific evidence from over 10000 studies confirms that e-cigarettes damage the respiratory and circulatory systems, are largely ineffective as cessation aids, and, critically, increase the likelihood of young users transitioning to conventional cigarettes^5^. E-cigarettes are now established as a health hazard, containing synthetic nicotine, free radicals, and chemicals that may potentially cause serious health conditions^6-11^.

Existing Thai research primarily investigates e-cigarette use among older groups, such as vocational students in Buriram^12^, undergraduate students in Phitsanulok^13^, and upper secondary students in Bangkok^14^. These studies found that peer influence, online marketing, and existing smoking habits are key factors. There remains a critical gap in research focusing on lower secondary school students (Grades 7–9). This group is highly vulnerable, often targeted by e-cigarette companies with appealing product designs and flavors, yet they lack adequate, evidence-based prevention campaigns^15^. Understanding the drivers of intention in this younger, pre-initiation group is crucial for effective primary prevention.

Therefore, this research aims to investigate the factors associated with the intention to use e-cigarettes among lower secondary school students in the Bangkok Metropolitan Area, a demographic (comprising factors such as gender, grade, and socio-economic background) identified as being at high risk of becoming new users. The study utilizes the Theory of Planned Behavior (TPB)^16^, which posits that human behavior is guided by three psychological constructs: attitudes, subjective norms, and perceived behavioral control. TPB provides a robust framework for assessing ‘intention’ by accounting for both personal beliefs and social influences that shape early decision-making in adolescents. The findings may provide useful, theory-driven evidence to inform the development of targeted anti-e-cigarette campaigns and prevention strategies for adolescents in similar metropolitan contexts.

## METHODS

### Study design and participants

This research employed a cross-sectional survey design, targeting lower secondary school students enrolled in schools under the Bangkok Metropolitan Administration (BMA), totaling 35641 students^17^ (A STROBE checklist is provided in the Supplementary file).

The required sample size of 300 students was calculated using the Krejcie and Morgan formula^18^ based on previously reported proportions of intention to use e-cigarettes^19^. The sample size of 300 was deemed sufficient for binary logistic regression analysis. This exceeds the minimum requirement of 10 observations per independent variable to ensure model stability^20^. Furthermore, considering the 11 independent variables (m=11) used in this study, the sample size satisfies the guideline of n >(50 + 8m), which requires a minimum of 138 participants for adequate statistical power^21^.

**Figure 1:**
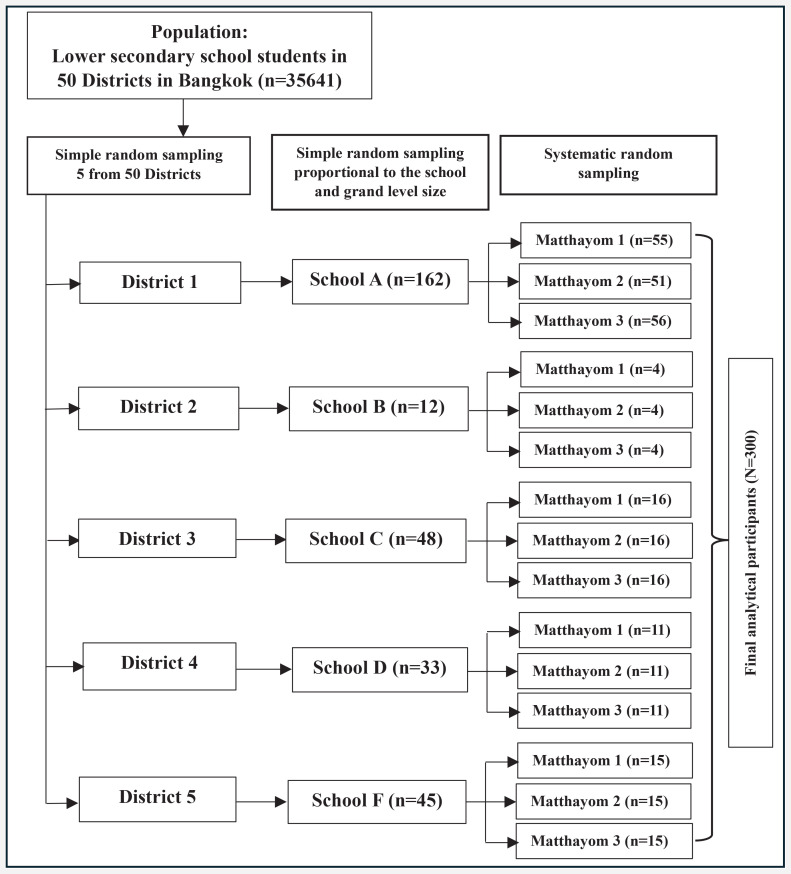
Flowchart of sample selection among lower secondary school students in Bangkok, 2024 (N=300)

The participants were recruited using a two-stage cluster random sampling technique. In the first stage, cluster sampling was used to select 5 districts from the 50 districts in Bangkok. In the second stage, a two-step simple random sampling process was conducted: first, to select one representative school per district; and second, to select one classroom per grade level within each selected school. Data collection was performed among students in these classrooms using systematic random sampling. The sample size for each school was determined based on probability proportional to size (PPS), as shown in Figure 1.

Inclusion criteria stipulated that participants must be students listed in the 2024 academic year enrollment records of BMA schools. Students who were absent during the data collection period were excluded. Data were collected between May and July 2024.

### Ethics

This study was approved by the Human Research Ethics Committee of Ramkhamhaeng University (Approval No. RU-HRE 67/0077; Date: 17 May 2024). Before data collection, the researchers coordinated with school administrators to send information sheets and consent forms to parents or legal guardians via the students two weeks in advance. Only students who provided written informed consent from both themselves and their parents were permitted to participate in the study.

### Variables

The research instrument employed was a self-administered questionnaire developed by the researcher to measure the study variables in relation to the Theory of Planned Behavior (TPB) constructs. The questionnaire consisted of five parts.

### Personal characteristics

This section collected essential personal and background information. The eight items included gender (male, female), age (years), current grade (Matthayom 1, 2, and 3), average daily allowance, living arrangement (alone, with family/relatives, with friends, or other), presence of underlying diseases (yes, no), and smoking history within the past year (never, ever; specifying type: conventional cigarette, e-cigarette, or other). It also assessed exposure to both positive and negative e-cigarette information via various media (e.g. peers, TV, internet, radio).

### Attitude toward e-cigarette prevention

This part measured the participant’s attitude regarding the outcomes and consequences of e-cigarette use. It contained 15 items presented on a 5-point Likert scale (strongly disagree to strongly agree). The operational definition of ‘attitude’ in this study encompasses two dimensions: 1) Social-psychological perceptions, such as social normalcy, appeal (coolness, wealth), and e-cigarettes as a cessation tool; and 2) Health-related beliefs, including risks like lung inflammation and heart disease.

To ensure the tool was contextualized for Thai adolescents, items included perceptions of vaping in local social settings (e.g. dining tables and entertainment venues) and the appeal of flavored aromas. Scores were categorized into low (1.00–2.33), medium (2.34–3.67), and high (3.68–5.00) using Best’s criteria^18^, which provide an objective class-interval based on the range of the Likert scale, ensuring a standardized classification regardless of minor score fluctuations.

### Subjective norms toward e-cigarette prevention

This section assessed subjective norms, focusing on the perceived social pressure or influence from important reference groups on the intention to use e-cigarettes. The 11 items utilized the same 5-point Likert scale. Questions addressed the influence of peers, older/younger students, relatives, parents, teachers, and media role models (e.g. celebrities/net idols) on the participant’s desire or intention to smoke e-cigarettes. The scores were categorized into high (3.68–5.00), medium (2.34–3.67), and low (1.00–2.33) levels based on Best’s criteria^18^.

The 11 items for subjective norms were specifically designed to capture the Thai adolescent social context, where interpersonal relationships with seniors, juniors, and ‘net idols’ (influential online figures) play a significant role in shaping behavior. These items assess whether e-cigarette use is perceived as a normative social practice (e.g. following admired peers or celebrities) or a deviant behavior that is discouraged by traditional authority figures such as parents and teachers. Items 7 and 9 were reverse-coded to ensure internal consistency in measuring the perceived social pressure toward e-cigarette use.

### Perceived behavioral control (PBC) for e-cigarette prevention

This part measured participants’ perceived behavioral control – their belief in their ability to control themselves from using e-cigarettes. The 13 items also used the 5-point Likert scale. Items included factors such as fear of bodily harm, legality concerns, social acceptance (fear of non-acceptance), cost, accessibility, and environmental controls (e.g. warning signs, school monitoring). The scores were categorized into high (3.68–5.00), medium (2.34–3.67), and low (1.00–2.33) levels using Best’s criteria^22^.

The 13 items for perceived behavioral control (PBC) were designed to assess both internal and external factors influencing the students’ ability to refrain from e-cigarette use. In the Thai educational context, internal factors include fear of academic punishment and the ‘fear of disappointing parents’ (filial piety), a strong cultural deterrent. External factors include the physical environment of Thai schools (e.g. presence of inspectors and warning signs) and the marketing appeal of e-cigarettes (e.g. aroma and packaging). Items 4, 5, 9, 10, 11, 12, and 13 were reverse-coded so that higher total scores reflected lower perceived control (or higher inclination toward the behavior) to remain consistent with other TPB constructs.

### Intention to use e-cigarettes

This final section measured the participants’ intention to engage in the behavior. It consisted of seven items using an 11-point numerical rating scale (0–10), where 0 represented ‘definitely not intend’, and 10 represented ‘definitely intend’. The items covered future intentions across different timeframes (e.g. ‘in the future’, ‘in the next 6 months’, ‘lifetime non-use’). Originally, these were interpreted using Bloom’s criteria^23^; the total scores (0–70) were originally categorized into three levels: low (0–41), moderate (42–55), and high (56–70). However, due to the highly skewed distribution of the data, the moderate and high groups were collapsed into a single ‘high intention’ category for the final analysis, resulting in a binary classification: low vs high intention. Intention to use e-cigarettes was measured using the following seven items: 1) I expect to use e-cigarettes in the future; 2) I expect to use e-cigarettes within the next 6 months; 3) I expect to use e-cigarettes within the next month; 4) I will not use e-cigarettes for the rest of my life; 5) I will not use e-cigarettes for the sake of my health; 6) I will not use e-cigarettes for my loved ones; and 7) I will not use e-cigarettes to be a good role model for my friends. Items 4 to 7 were reverse-coded before data analysis so that higher total scores consistently represented a stronger intention to use e-cigarettes.

The instrument’s content validity was established through expert review by three specialists using the Index of Item-Objective Congruence (IOC), with scores ranging from 0.67 to 1.00, indicating high content validity. Internal consistency was subsequently evaluated via a pilot study conducted with 30 students from a school in the same province; these participants shared similar characteristics with the target population but were not included in the final sample. The results demonstrated acceptable to good reliability across all constructs: attitude toward e-cigarette prevention (α=0.71), subjective norms (α=0.85), perceived behavioral control (α=0.84), and intention to use e-cigarettes (α=0.74). Following the pilot test, the researchers coordinated with participating schools to establish a collection schedule. The onsite survey was conducted in classrooms or designated multi-purpose halls. To minimize potential bias and social desirability effects, teachers were asked to remain at the front of the room or temporarily exit the area while researchers explained the questionnaire and supervised the process. This ensured that students could answer honestly without fear of academic repercussions.

### Statistical analysis

The survey data were analyzed using SPSS (version 18.0: copyright Mahidol University). Descriptive Statistics were employed to summarize the personal characteristics and TPB constructs (attitude toward e-cigarette prevention, subjective norms, PBC, and intention to use e-cigarettes). Categorical variables are presented as frequencies and percentages, while continuous variables are described as mean, standard deviation, and range.

TPB constructs were initially categorized into three levels (low, moderate, and high) for descriptive purposes. However, due to a highly skewed distribution with a predominant ‘low intention’ category, the ‘moderate’ and ‘high’ levels were collapsed into a single group for binary logistic regression. This adjustment ensured sufficient statistical power and prevented empty cell issues in contingency tables.

Bivariate analysis was conducted to examine differences between the intentional and non-intentional groups: the chi-squared test was used for categorical variables, and the independent t-test was used for continuous TPB constructs.

Subsequently, binary logistic regression was performed using a two-step approach: First, an unadjusted model (crude odds ratio, OR) was used to identify potential predictors. In this stage, variables that exhibited a p<0.25 in the bivariate analysis were selected as candidates for the next step. Second, an adjusted model (multivariable logistic regression) was constructed, where these selected variables were entered simultaneously. This allowed for the calculation of adjusted odds ratios (AOR) with 95% confidence intervals (CIs) while controlling for potential confounders. For the final multivariable model, a p<0.05 was considered statistically significant.

## RESULTS

### Personal characteristics of participants

This study recruited a total of 300 lower secondary education students in Bangkok, comprising a sample that was predominantly female (59.0%) with an average age of 13.6 years (SD=0.9). The sample was drawn mainly from Grade 9 (Matthayom 3) (34.0%), and the vast majority (98.3%) reported living with family or relatives. Financially, most students received a daily allowance of 100–149 THB. Furthermore, 13.3% of the participants reported a chronic illness, with allergies being the most common condition (50.0%). Regarding the prevalence of tobacco and e-cigarette use, the study found that 85 participants (28.3%) reported a history of smoking within the past year. Among those who had ever smoked, e-cigarettes were the most common product used (88.0%), followed by conventional cigarettes (54.1%). For sources of information on e-cigarettes, participants primarily received information about e-cigarettes through digital platforms, with the internet and social media (e.g. Facebook, TikTok) being the most common sources at 82.0%. Interpersonal sources, such as friends and teachers, accounted for 66.0%, while conventional media, such as television, reached 47.0% of students, as shown in Table 1.

**Table 1:** General characteristics and e-cigarette information exposure of lower secondary students in Bangkok, 2024 (N=300)

*Characteristics*	*n*	*%*
**Gender**		
Male	123	41.0
Female	177	59.0
**Age** (years)		
12–14	246	82.0
15–17	54	18.0
**School grade**		
7	101	33.7
8	97	32.3
9	102	34.0
**Daily allowance** (THB)		
<50	16	5.3
50–99	111	37.0
100–149	145	48.3
≥150	28	9.4
**Living status**		
Living alone	4	1.3
Living with others	296	98.7
**Underlying diseases**		
No	260	86.7
Yes	40	13.3
**Type of diseases** (n=40)*		
Allergies	20	50.0
Asthma	12	30.0
G6PD deficiency	3	7.5
Thyroid disease	2	5.0
Anemia	2	5.0
Hypertension	1	2.5
**Tobacco use history in the past year**		
Never	215	71.7
Ever	85	28.3
**Type of tobacco products** (n=85)*		
E-cigarettes/vaping products	75	88.0
Manufactured cigarettes	46	54.1
Other tobacco products	1	1.2
Sources of information on e-cigarettes*		
Social media and internet (e.g. Facebook, TikTok)	246	82.0
Interpersonal sources (e.g. friends, teachers)	198	66.0
Television	141	47.0
Radio	11	3.7
Other	1	0.3

* Respondents could select more than one option. THB: 1000 Thai Baht about US$31.

### Results based on the Theory of Planned Behavior (TPB)

According to the Theory of Planned Behavior, the analysis of TPB constructs among lower secondary school students in Bangkok revealed the following:

*Attitude toward e-cigarette prevention*

The majority of participants reported a high level of preventive attitude (69.7%). The most strongly held perception was that vaping makes one look ‘cool or wealthy’, while the belief that vaping causes poor memory was the least emphasized.

*Subjective norms toward e-cigarette prevention*

Most participants exhibited a high level (75.3%). The primary social influence was the desire to be perceived as cool or wealthy by others. In contrast, the influence of teachers’ warnings was the weakest reported factor among the reference groups.

*Perceived behavioral control (PBC) for e-cigarette prevention*

A significant majority reported a high level of self-control (81.7%). The strongest factor in their perceived control was again related to social image (coolness and wealth), while the high cost of e-cigarettes was the least significant factor influencing their perceived control over use.

*Intention to use e-cigarettes*

Regarding the intention to use e-cigarettes, the vast majority of students reported a low level (90.7%), followed by moderate (6.0%) and high (3.3%). The highest commitment was found in the intention to remain smoke-free for a lifetime, while the lowest intention was reported for using e-cigarettes within the next month, as shown in Table 2.

**Table 2:** Levels of Theory of Planned Behavior (TPB) factors related to e-cigarette use among lower secondary school students in Bangkok, 2024 (N=300)

*Variables*	*n (%)*			*Mean*	*SD*	*Median*	*Range*
	*Low*	*Moderate*	*High*				
**Attitudes toward e-cigarette prevention**	0 (0.0)	91 (30.3)	209 (69.7)	4.0	0.5	4.0	2.5–5.0
**Subjective norm**	8 (2.7)	66 (22.0)	226 (75.3)	4.3	0.8	4.5	1.6–5.0
**Perceived behavioral control**	3 (1.0)	52 (17.3)	245 (81.7)	4.2	0.7	4.4	1.5–5.0
**Intention**	272 (90.7)	18 (6.0)	10 (3.0)	16.0	18.2	8	0–70

Theory of Planned Behavior factors level (Items 2.1-2.3): Low 1.00–2.33, Moderate 2.34–3.66, High 3.67–5.00. Intention level: Low 0–41, High 42–70 score.

### Bivariate analysis

Bivariate analysis was conducted to examine the factors associated with the intention to use e-cigarettes. Comparison of TPB construct scores using Pearson’s chi-squared and independent t-tests showed significant differences between the intentional and non-intentional groups (Table 2). Subsequent univariate binary logistic regression indicated that gender was a statistically significant factor (OR=2.42; 95% CI: 1.09–5.37, p=0.030). Regarding TPB constructs, two variables demonstrated a statistically significant influence on intention to use e-cigarettes at the p<0.05 level: attitude towards e-cigarette prevention (OR=4.14; 95% CI: 1.85–9.24, p<0.001) and perceived behavioral control (OR=2.88; 95% CI: 1.30–6.65, p=0.013). Additionally, subjective norms also demonstrated a notable influence, meeting the less restrictive threshold of p<0.25 for inclusion in a subsequent multivariate model (OR=2.15; 95% CI: 0.96–4.82, p=0.064) (Table 3).

**Table 3:** Comparison of personal characteristics between intentional and non-intentional groups among lower secondary school students in Bangkok, 2024 (N=300)

*Variables*	*n (%)*	*Intention level n (%)*		*χ^2^/t*	*p*
		*Low*	*High*		
**Total**	300 (100)	272 (90.7)	28 (9.3)		
**Gender**					
Male	123 (41.0)	106 (86.2)	17 (13.8)	4.962	0.026*
Female	177 (59.0)	166 (93.8)	11 (6.2)		
**Age** (years)					
12–14	246 (82.0)	224 (91.1)	22 (8.9)	-1.812	0.071
15–17	54 (18.0)	48 (88.9)	6 (11.1)		
Mean ± SD	13.6 ± 0.9	13.6 ± 0.9	13.9 ± 0.7		
Range	12–16	12–16	13–15		
**School grade**					
7	101 (33.7)	94 (93.1)	7 (6.9)	5.304	0.071
8	97 (32.3)	91 (93.8)	6 (6.2)		
9	102 (34.0)	87 (85.3)	15 (14.7)		
**Daily allowance** (THB)					
<50	127 (42.3)	116 (91.3)	11 (8.7)	-0.048	0.931
≥50	173 (57.7)	156 (90.2)	17 (9.8)		
Mean ± SD	95.6 ± 72.5	95.6 ± 75.3	96.3 ± 35.1		
Range	20–1200	20–1200	40–160		
**Living status^a^**					
Living alone	4 (1.3)	3 (75.0)	1 (25.0)	-	0.326
Living with others	296 (98.7)	269 (90.9)	27 (9.1)		
**Underlying diseases^a^**					
No	260 (86.7)	236 (90.8)	24 (9.2)	-	0.776
Yes	40 (13.3)	36 (90.0)	4 (10.0)		
**Tobacco use history in the past year**					
Never	215 (71.7)	197 (91.6)	18 (8.4)	0.829	0.382
Ever	85 (28.3)	75 (88.2)	10 (11.8)		

Intention level: Low 0–41, High 42–70. THB: 1000 Thai Baht about US$31.

### Multivariate analysis

The multivariate analysis showed that students with a low attitude toward e-cigarette prevention were more likely to have an intention to use e-cigarettes (AOR=3.82; 95% CI: 1.40–10.39, p=0.009). Specifically, students with less favorable attitudes were 3.82 times more likely to report an intention to use e-cigarettes compared to those with more favorable attitudes, holding other factors constant. Conversely, other factors in the model, including gender, subjective norm, and perceived behavioral control, did not show statistically significant associations with e-cigarette use intention (p>0.05). Collectively, the four variables included in the model explained 11.3% of the variance in e-cigarette use intention (Table 4).

**Table 4:** Univariable and multivariable analysis of factors associated with e-cigarette intention among lower secondary school students in Bangkok, 2024 (N=300)

*Variables*	*Univariate analysis*		*Multivariate analysis*			
	*OR (95% CI)*	*p*	*β*	*SE*	*AOR (95% CI)*	*p*
**Personal factors**						
**Gender**						
Male	2.42 (1.09–5.37)	0.030*	0.749	0.418	2.12 (0.93–4.80)	0.073
Female (ref.)	1				1	
**Age** (years)						
12–14 (ref.)	1					
15–17	1.27 (0.49–3.31)	0.621				
**School grade**						
7–8 (ref.)	1					
9	1.58 (0.65–3.86)	0.312				
**Daily allowance** (THB)						
<100 (ref.)	1					
>100	0.87 (0.40–1.93)	0.732				
**Living status^a^**						
Living alone (ref.)	1					
Living with others	3.32 (0.34–33.04)	0.306				
**Underlying diseases^a^**						
No (ref.)	1					
Yes	1.09 (0.36–3.33)	0.876				
**Tobacco use history in the past year**						
Never (ref.)	1					
Ever	1.46 (0.64–3.31)	0.365				
**Theory of Planned Behavior (TPB) factors**						
**Attitudes toward e-cigarette prevention**						
Low	-	-	-	-	-	-
Moderate	4.14 (1.85–9.24)	<0.001***	-1.340	0.511	3.82 (1.40–10.39)	0.009
High (ref.)	1	1				
**Subjective norm**						
Low	2.15 (0.96–4.82)	0.064^a^	-0.265	0.563	0.77 (0.26–2.31)	0.638
Moderate/High (ref.)	1	1				
**Perceived behavioral control**						
Low	2.88 (1.3–6.65)	0.013*	0.321	0.556	1.38 (0.46–4.10)	0.564
Moderate/High (ref.)	1	1				

Constant= -3.229, R^2^=0.113, p<0.05. SE: standard error.

a Variables with p<0.25 in the univariable analysis were included in the multivariable logistic regression model. The variance inflation factor (VIF) for all independent variables was <1.63, indicating no significant multicollinearity. Theory of Planned Behavior factors level (Item 2.1-2.3): Low 1.00–2.33, Moderate 2.34–3.66, High 3.67–5.00. Intention level: Low 0–41, High 42–70.

* p<0.05.

** p<0.01.

*** p<0.001.

## DISCUSSION

The results indicate that the majority of lower secondary students in Bangkok (90.7%) have a low intention to use e-cigarettes and do not plan to use them in the near future. Only a small fraction reported uncertainty regarding lifetime non-use. This finding is consistent with prior studies conducted in Thailand, such as the research by Konkaew and Konkaew^19^ among Northern Thai youth, which reported a low intention rate of 74.5%, and the study by Patiño-Masó^24^, which indicated that 86.3% of high school students reported no intention to use e-cigarettes in the future. This high level of non-intention may be reinforced by Thailand’s strict legal framework, which prohibits the import, sale, and possession of e-cigarettes. Such regulations likely contribute to the perception of vaping as a deviant or illicit behavior among students. However, the 9.3% ‘at-risk’ group remains a public health concern, especially as recent evidence suggests that ‘non-intenders’ can quickly transition to users when exposed to aggressive digital marketing and flavored products^25^. Furthermore, our study identified a vaping prevalence of 28.3% within the past year among this demographic, with e-cigarettes being the primary choice (88.0%) over conventional cigarettes. These findings underline the high prevalence of vaping among lower secondary school students in Bangkok, highlighting it as a significant public health concern for this age group that requires immediate attention.

A noticeable gender disparity was observed, with male students exhibiting a significantly higher proportion of intention to use e-cigarettes compared to their female counterparts (13.8% vs 6.2%). Beyond sensation-seeking, this disparity may be reinforced by the ‘techno-cognitive’ appeal of e-cigarette devices, which are often marketed as high-tech gadgets that appeal more to male adolescents^26^. In the Thai context, this is exacerbated by social norms that still perceive nicotine use as a masculine trait, despite the rising prevalence among females^27^. These findings are consistent with established domestic and international literature, including studies by Konkaew and Konkaew^19^ in Thailand and Zou et al.^28^. This male predominance in both China and Thailand suggests that nicotine initiation is often tied to traditional concepts of masculinity and risk-taking behavior in Asian cultures, which differs from the more gender-neutral trends observed in some Western European studies.

Regarding attitudes toward e-cigarette prevention, most students demonstrated strong support (69.7%). Crucially, students with higher prevention-related attitude scores were significantly less likely to intend to use e-cigarettes (AOR=0.26; 95% CI: 0.10–0.71) compared to those with lower scores. This finding strongly supports the tenets of the Theory of Planned Behavior (TPB)^16^, which posits that an individual’s beliefs about the outcomes of a behavior influence their attitudes, thereby shaping their behavioral intention. While Auemaneekul et al.^29^ emphasized fear as a deterrent, our results suggest that internalizing health risks is a more stable predictor of long-term abstinence. This contrast is evident when comparing our youth sample to that in Northern Thailand^30^ and the American adult study^31^. The difference between our findings and the American study^31^ highlights the developmental gap; while adults may prioritize long-term health risks, adolescents are more driven by immediate social rewards and digital imagery, necessitating prevention strategies that focus on social identity rather than just health warnings.

For subjective norms, the majority of students (75.3%) reported high subjective norms toward e-cigarette prevention. Notably, a subset of students demonstrated lower compliance with teachers’ warnings against e-cigarette experimentation. Specifically, our analysis of subjective norm sub-items revealed that (24.7%) of students reported a low intent to comply with school authorities. This suggests that institutional authority may be losing its influence compared to the ‘modern’ social norms promoted by digital peers. Statistical analysis revealed that subjective norms against vaping were significantly associated with a lower intention to use e-cigarettes among participants. This finding aligns with the Theory of Planned Behavior (TPB), which posits that an individual’s behavioral intention is heavily influenced by the perceived social pressure from significant others^16^. This is supported by Patiño-Masó et al.^24^ who found that the predictors of the intention to use electronic cigarettes in the future, were having friends who smoke or use electronic cigarettes. Thus, parental and peer behaviors, as noted by Summat and Ruenphet^11^, Alber et al.^30^ and Scheinfeld et al.^32^ remain the primary drivers of the social environment. In the specific context of Thai adolescents, these subjective norms are further complicated by the ‘seniority’ system (seniors/juniors) and the pervasive influence of ‘net idols’ on social media platforms used by 82.0% of our participants to access e-cigarette information. When vaping is portrayed by admired peers or digital influencers as a trendy and modern lifestyle choice, it shifts the perception of e-cigarette use from a ‘deviant behavior’ discouraged by traditional authorities to a ‘normative social practice’ within youth circles. This explains the observed lower compliance with teachers’ warnings, as the social pressure to fit into a perceived modern identity may outweigh the influence of academic or parental figures in an urban environment like Bangkok^33^.

For perceived behavioral control, students reported high perceived behavioral control (PBC) (81.7%), which was significantly and negatively associated with e-cigarette use intention. However, this relationship was attenuated and did not reach statistical significance in the multivariate model. This suggests that a strong sense of self-efficacy in resisting e-cigarettes functions as a protective barrier against initiation^16^; its individual impact is likely overshadowed by other factors like attitudes or social norms when all variables are considered simultaneously. Specifically, refusal self-efficacy – the students’ confidence in their ability to decline offers from peers – acts as a crucial cognitive resource that strengthens this protective barrier, especially in high-pressure social situations. Interestingly, our findings partially contrast with some international literature. For instance, while studies by Sutherland et al.^34^ and Phipps et al.^35^ found PBC to be a robust predictor of abstinence, other research among Asian youth has shown that collective social influences and environmental triggers – such as easy access to products^19^ and social acceptance within peer groups^26^ – can override individual self-control^26,30^. Furthermore, some studies have found that peer influence outweighs individual attitudes^32^, which differs from our results, where attitudes remained the strongest predictor. These discrepancies may be attributed to the ‘normalization’ of vaping in Bangkok’s urban public spaces^33^, which may weaken the protective effect of an individual’s perceived control. Therefore, while strengthening students’ behavioral control remains a potential area for intervention, programs may benefit more from prioritizing the shift in internalized attitudes and social identity.

In summary, e-cigarette use intention among lower secondary students is low, yet significantly higher in males. The study confirms that, consistent with the Theory of Planned Behavior (TPB), attitude toward prevention and high perceived behavioral control (PBC) are the most critical determinants of intention. These findings necessitate the implementation of gender-specific interventions focused on correcting misinformation to strengthen anti-vaping attitudes and delivering skills-based training to reinforce students’ PBC against e-cigarette use.

### Practical implications and recommendations

Based on the study’s findings, anti-e-cigarette campaigns for students and adolescents may benefit from focusing on strengthening positive preventive attitudes. Interventions could consider incorporating digital media literacy to help students critically evaluate online vaping content, especially on platforms like TikTok and Facebook. Given that attitude was the most consistent predictor in the multivariate analysis, strategies aimed at internalizing health risks and social identity might be more effective than traditional fear-based messaging. Furthermore, programs could support the development of refusal self-efficacy as a secondary protective factor, providing students with the specific behavioral skills and confidence needed to navigate peer pressure. Finally, interventions should consider leveraging influential social agents – specifically, peers and parents – to promote a ‘non-vaping’ social norm that counters the current normalization of e-cigarettes in urban public spaces.

### Limitations

This study has several limitations. First, the urban Bangkok setting may limit generalizability to rural areas. Second, the cross-sectional design precludes causal inferences. Third, the illegal status of e-cigarettes may induce social desirability bias. Fourth, the gender imbalance (59% female) may introduce selection bias. Fifth, self-reporting is subject to recall bias. Additionally, the low R^2^ (11.3%) suggests residual confounding by unmeasured variables. Finally, non-response bias may exist, as absent students – potentially a higher-risk group – were excluded. Future research should consider longitudinal designs and psychometric validation (e.g. confirmatory factor analysis).

## CONCLUSIONS

The findings suggest that lower secondary school students in Bangkok generally have a low level of intention to use e-cigarettes. Gender was associated with intention, with male students exhibiting higher rates. Consistent with the multivariate results of the Theory of Planned Behavior, attitude toward prevention emerged as a significant predictor of intention. These results suggest the importance of focusing on anti-vaping attitudes within prevention programs for urban youth.

## Supplementary Material



## Data Availability

The data supporting this research are available from the authors on reasonable request.
